# Electrospun Rubber Nanofiber Web-Based Dry Electrodes for Biopotential Monitoring

**DOI:** 10.3390/s23177377

**Published:** 2023-08-24

**Authors:** Mohammad Shamim Reza, Lu Jin, You Jeong Jeong, Tong In Oh, Hongdoo Kim, Kap Jin Kim

**Affiliations:** 1 Department of Advanced Materials Engineering for Information & Electronics, Kyung Hee University, Yongin 17104, Gyeonggi-do, Republic of Korea; shamimtex2012@gmail.com (M.S.R.); jinlu1011@hotmail.com (L.J.); 2 Department of Biomedical Engineering, School of Medicine, Kyung Hee University, Seoul 02447, Republic of Korea; yj.jeong0107@gmail.com (Y.J.J.); tioh@khu.ac.kr (T.I.O.)

**Keywords:** electrospinning, nanofiber web, silver plating, dry electrode, biopotential monitoring

## Abstract

This study aims to find base materials for dry electrode fabrication with high accuracy and without reducing electrode performance for long-term bioelectric potential monitoring after electroless silver plating. Most applications of dry electrodes that have been developed in the past few decades are restricted by low accuracy compared to commercial Ag/AgCl gel electrodes, as in our previous study of PVDF-based dry electrodes. In a recent study, however, nanoweb-based chlorinated polyisoprene (CPI) and poly(styrene-*b*-butadiene-*b*-styrene) (SBS) rubber were selected as promising candidates due to their excellent elastic properties, as well as their nanofibril nature, which may improve electrode durability and skin contact. The electroless silver plating technique was employed to coat the nanofiber web with silver, and silver nanoweb(AgNW)-based dry electrodes were fabricated. The key electrode properties (contact impedance, step response, and noise characteristics) for AgNW dry electrodes were investigated thoroughly using agar phantoms. The dry electrodes were subsequently tested on human subjects to establish their realistic performance in terms of ECG, EMG monitoring, and electrical impedance tomography (EIT) measurements. The experimental results demonstrated that the AgNW dry electrodes, particularly the SBS-AgNW dry electrodes, performed similarly to commercial Ag/AgCl gel electrodes and were outperformed in terms of long-term stability.

## 1. Introduction

Endogenic biopotentials are attractive signals to monitor physiological and pathological phenomena, since they can be measured continuously and non-invasively [1,2]. Due to the ease of measurement by the electronic system, the electrical properties inside a body can be monitored by applying electric energy outside the body and measuring the modulated exogenic electric potentials [3]. In both cases, electrodes are essential for measurement. Moreover, the measurement quality of bioelectrical potentials is highly dependent on the electrodes involved [4,5]. Therefore, a particular type of electrode is required in ubiquitous healthcare systems, brain computing interface (BCI) and human–computer interface (HCI) techniques, and bionic engineering depending on the kinds of biopotential to be measures and the measurement position [4,6,7,8,9,10,11,12,13,14,15]. Most ubiquitous healthcare systems and BCIs measure physiological signals, such as electrocardiography (ECG) and electroencephalography (EEG), to monitor the human body and control devices [11,12,16]. Electromyography (EMG) is generally used to diagnose muscle disease or/and muscle activities, and it can also be employed to handle artificial limbs in bionic engineering [10]. Unlike measuring endogenous biopotentials, electrical impedance tomography (EIT) applies high-frequency AC electric signals to the body through multiple electrodes [17]. It requires a broader frequency range of electrodes, and the used electrodes must have similar characteristics. Therefore, the development and performance evaluation of electrodes that can measure bioelectric potentials with various frequency characteristics and amplitudes are critical factors.

Ag/AgCl gel electrodes are commonly used for clinical biopotential recording since they offer a close approximation to nonpolarizable electrodes and their half-cell potential is highly compatible with body composition. Ag/AgCl gel electrodes could provide signal fidelity to measure ECG, EEG, EMG, and EIT because they have been well-characterized, medically verified, and studied for many decades [5,6,7,8,9,10,11,12,13,14,15,16,17,18,19]. However, it is difficult to apply Ag/AgCl gel electrodes in long-term monitoring, since the performance of the electrode can be degraded over time as the gel in the conductive gel pad of the Ag/AgCl electrode dries. Additionally, they can cause skin irritation and be uncomfortable to remove from the skin due to the strong adhesiveness of one side [4,6,7,9,10,19,20,21].

Therefore, many studies have been conducted to develop new electrodes and evaluate them using Ag/AgCl gel electrodes as a standard for comparison. In the last decade, including a few recent reports by Kisannagar et al. [22], Alizadeh-Meghrazi et al. [23], and Voica et al. [24], various types of electrodes without any gel, named dry electrodes, have been reported to overcome the drawbacks of Ag/AgCl gel electrodes [5,7,19,25,26,27,28,29,30,31,32,33,34]. They are roughly classified into two categories: metal-based electrodes and polymer-based electrodes. Previously, metal-based electrodes were reported in the form of metallic suction type, needle form with rigid metal pins [6], steel plate [35] type, etc. Solid metal dry electrodes were well documented to cause discomfort and trigger significant motion artifacts as a result of electrode slippage [11,19,20,21,36]. Conversely, polymer-based electrodes can be classified into two subtypes: composite (foam or rubber-based) electrodes [6,7,37] and textile/fabric electrodes [4]. Polymer-based electrodes have attracted attention with respect to comfort and usability because they are flexible and can quickly adapt to skin topography while providing proper contact with curvy skin. However, one of the main limitations of those polymer (foam/sheet) composite electrodes is that they have relatively high electrical resistance because they are generally fabricated with the addition of conductive additives in the polymers to endow them with electric conductivity [9,10,21,22,23,37,38]. The performance of textile/fabric electrodes is degraded by surface abrasion, poor coating durability, and variations in electric performance with repeated usage [4,20,39]. Oh et al. reported several kinds of fabric-based dry electrodes, including two types of electrospun nanofiber web-based electrodes: PEDOT-coated PVDF and silver-plated PVDF nanofiber web-based electrodes [4,9]. The authors reported that silver-plated nanoweb-based electrodes revealed better intrinsic properties than fabric-based electrodes. However, one downside of PVDF nanowebs is their poor durability due to their low elasticity, which makes handling difficult when detaching the nanoweb from the collecting substrate for subsequent silver-plating procedures, which reduces the electrode quality [40].

In the present work, we introduced chlorinated polyisoprene electrospun silver-plated nanoweb-based dry electrodes (CPI-AgNW) for the first time. Chlorinated polyisoprene, a highly elastomeric material, is made by solution polymerization of isoprene (2-methyl-l,3-butadiene) followed by chlorination. Lenko et al. showed that the hydrophilicity of the polyisoprene rubber surface increased significantly after chlorination [41]. On the other hand, poly(styrene-*b*-butadiene-*b*-styrene) is a triblock copolymer that consists of rigid styrene and soft butadiene segments, which makes it highly elastic, with excellent rubbery properties; it is also much more durable and inexpensive than PVDF. In consideration of easy and uniform silver plating on the nanoweb surface, CPI and SBS are more favorable than PVDF, since the rubber polymer-based nanoweb is more hydrophilic than PVDF, which plays a vital role in the silver-plating process.

Our prior work concentrated on TPU and SBS rubber nanofiber web-based dry electrodes for vital signal monitoring. However, the SBS rubber dry electrode was not characterized in detail. The obtained ECG waveform fidelity of TPU nanoweb-based dry electrodes did not satisfy our expectations, as it was about 93% of that obtained with Ag/AgCl gel electrodes, and the intrinsic electrode properties, i.e., noise characteristics of the SBS dry electrode, were not at a satisfactory level compared to those of the Ag/AgCl gel reference electrode [4,40].

Consequently, further study is required with a top-to-bottom optimized electrode fabrication technique using a unique and highly precise ultrafine and thin nanofiber web structure through the optimized electrospinning of CPI and SBS rubber and step-by-step upgraded electroless silver-plating technique with a uniform and highly electrically conductive Ag-nanoweb surface to achieve relatively superior electrode properties. Intrinsic electrode parameters such as contact impedance, step response, noise characteristics (for SBS-AgNW dry electrodes), and the realistic performance of AgNW dry electrodes were fully examined in this work. Human subject testing was performed with a volunteer using ECG and EMG for hand and leg muscle activities and EIT measurements for respiration monitoring of the SBS-AgNW dry electrodes, which has not been previously reported. The ECG and EMG signal fidelities of AgNW dry electrodes were determined to be 99% and 100%, respectively, of that of clinically verified Ag/AgCl gel electrodes, which is significantly superior compared to previously reported TPU nanoweb dry electrodes. In the present study, we also conducted a comparative electrode stability evaluation for long-term ECG monitoring of human subjects, with the findings demonstrating that the SBS-AgNW dry electrodes outperform Ag/AgCl gel electrodes.

## 2. Materials and Methods

### 2.1. Preparation of Nanofiber Web, Electroless Silver Plating, and Fabrication of Dry Electrode

Poly(styrene-*b*-butadiene-*b*-styrene) SBS chip with 31 wt. % styrene content (LG Chem. Ltd., Seoul, Republic of Korea) was used in this study. A solution of 14 wt. % (*w*/*v*) SBS was prepared by dissolving in a ternary solvent system (THF, Aldrich, USA/toluene, Alfa Aesar^®^, Haverhill, MA, USA/DMF, Aldrich, St. Louis, MI, USA (5/3/2 *v*/*v*/*v*)) [16]. Chlorinated polyisoprene (CPI, Mw = 450,000 (Approx.) (Scientific Polymer Products, Inc., Ontario, NY 14519, USA)) electrospun solution was prepared by dissolving 9 wt. % (*w*/*v*) CPI in a 3/2 (*v*/*v*) DMF/THF mixture. Silver-doped SBS and CPI solutions were prepared by adding a tiny amount of silver nitrate (Dae Jung, Busan, Republic of Korea) into SBS and CPI solution, respectively, at 1% based on the weight of the polymer and stirred for 24 h to form silver nanoparticles (Ag-NPs). The nanoparticles were formed on the nanofiber web surface from AgNO_3_ using DMF during electrospinning since the solvent is appropriate for the reduction process [42]. The key point of fabricating AgNW is that the reduced Ag-NPs on the nanofiber surface can act as catalysts to promote silver plating on the nanofiber surface. The electrospinning setup comprised a syringe pump, needle, high-voltage power supply, and drum collector. More details on electrospinning parameters and the silver-plating technique of electrospun nanowebs are provided in Supplementary Information, S1 Methodology.

### 2.2. Electrode Properties on Agar Phantom

After the fabrication of nanofiber web-based electrodes, three individual electrical properties (contact impedance, step response, and noise characteristics) of fabricated rubber-AgNW dry electrodes were assessed by utilizing an agar phantom with our established method [4,6,40]. We compared all electrode performances to those of typical Ag/AgCl gel (3M Red Dot Foam Monitoring ECG Electrodes 2237, USA Medical and Surgical Supplies, St. Louis, MO, USA) electrodes. During these assessments, all gel in the Ag/AgCl gel electrodes was removed for quantitative comparison with dry electrodes in the agar phantom (see Supporting Information, S2 Characterization).

### 2.3. Biopotential Recording on a Human Subject

**Figure 1:**
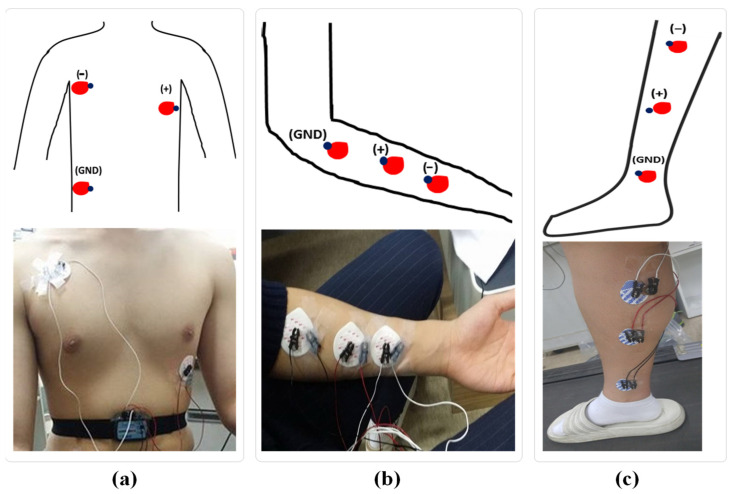
Schematic and photographic representations of the electrode setup on a human subject for biopotential recording. Red dots denote Ag/AgCl gel electrodes, and blue dots refer to AgNW dry electrodes. ECG measurement (**a**); EMG measurement for hand muscle activities (**b**); EMG measurement for leg muscle activities (**c**).

To compare the realistic performance of rubber-AgNW dry electrodes with that of Ag/AgCl gel electrodes, human volunteer tests were carried out on the body for ECG and EMG (hand and leg muscle activities). Bioelectric potentials were recorded simultaneously using both rubber-AgNW dry electrodes and Ag/AgCl gel electrodes, and the two types of electrodes were aligned as closely as possible on human subjects to minimize the error caused by position. A small adhesive part of the Ag/AgCl gel electrode was removed without the removal of the gel. A minimum gap of at least 20 mm between the Ag/AgCl gel electrode gel and the AgNW dry electrode was maintained to avoid electrical shortages between the two adjacent electrodes. Utilizing an adhesive tape (3M Micropore Medical Tape), AgNW dry electrodes were affixed to the subject. Figure 1 illustrates the positions of the electrodes used for various biopotential recordings.

Three AgNW dry electrodes and three Ag/AgCl gel electrodes were positioned in pairs on the subject’s chest for ECG recording, as shown in Figure 1a [6]. A commercially proven wireless ECG data acquisition module, BN-ECG2 (BioNomadix, Biopac Systems, Goleta, CA, USA), combined with an MP150 instrument (Biopac Systems, USA), was used as the ECG recording system. A fixed gain of 2000 and a bandpass filter with bandwidths of 1 Hz~35 Hz were employed. It should be noted that if electrodes are to be utilized for ECG biopotential monitoring, their performance over the approximately 0~250 Hz ECG bandwidth is of the utmost importance [4]. The subject should be sitting on a chair without movement during the test. Using a cross-covariance function in the MATLAB environment, the correlation between the two signals was computed based on the raw ECG signals recorded through both kinds of electrodes.

For EMG recording, the electrodes were affixed to the subject’s left forearm in pairs to observe hand muscle activities and the left leg in the tibia and fibula region to observe leg muscle activities, as shown in Figure 1b and c, respectively. A wireless EMG data acquisition module, BN-EMG2 (BioNomadix, Biopac Systems, USA), in conjunction with an MP150 instrument, was used to gather EMG signals. In this case, a fixed gain of 2000 with bandwidths of 10~500 Hz was employed. The subject should repeatedly clench and expand his left hand to generate muscle activities in the hand throughout the test. In contrast, leg muscle activities were recorded under three conditions: standing position (without walking) and walking at speeds of 3 km/h and 6 km/h observe slow and fast repeated leg muscle activities. Similarly, the correlations between the two EMG signals and the enveloped raw EMG signals were computed and compared.

### 2.4. EIT System and Imaging Experiments

**Figure 2:**
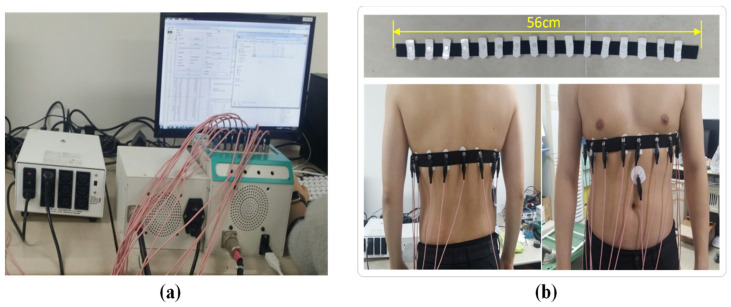
Experimental setup for EIT test: KHU Mark2 mfEIT system (**a**) [43] and an elastic belt with 16 electrodes attached and the band placed on the subject’s chest (back and front view) (**b**).

When electric energy is applied from the outside to measure electrical properties inside the body, a high-frequency current or voltage that cannot be generated in the human body is applied through the electrode, and the induced voltage or modified current is measured. We attempted to evaluate the performance of electrodes for a high-frequency electrical potential measurement by imaging two lungs whose electrical properties are changed by respiration in the thorax. The KHU Mark2 mfEIT system [43], as shown in Figure 2a, operates at 10 kHz, and an elastic belt made of an elastic band with a length of 56 cm and a width of 3 cm was adopted. The EIT belt contains 16 metal female eyelet connectors placed evenly from the center to connect different types of electrodes. One elastic belt was connected to 16 Ag/AgCl gel electrodes, and another was attached to 16 SBS-AgNW dry electrodes. Each belt was placed on the subject’s 5th intercostal plane, as shown in Figure 2b. The exogenic biopotentials induced by the applied high-frequency current were measured on multiple electrodes at 50 frames/s, repeating inspiration and expiration. Time difference impedance images were reconstructed by applying the GREIT algorithm with reference to the data obtained at the maximum expiration time.

## 3. Results and Discussion

### 3.1. Morphology of Electrospun Nanoweb before and after Silver Plating

The electrospinning process fabricated relatively uniform nanofibers. Beads or agglomerated nanofibers could not be observed in the obtained SBS and CPI nanoweb due to the high dielectric constant of the SBS and CPI solutions used in electrospinning. The diameter of the CPI nanofiber is considerably larger (≈571 nm) than the diameter of the SBS nanofiber (≈182 nm), mainly due to the variations in applied voltage and solution flow rates during electrospinning.

**Figure 3:**
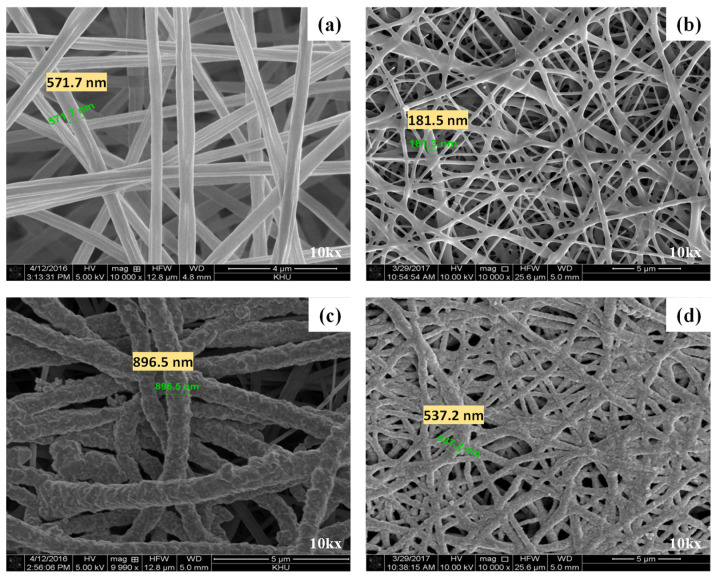
FIB-SEM images of as-spun CPI (**a**), as-spun SBS (**b**), silver-plated CPI (**c**), and silver-plated SBS (**d**) nanofiber webs.

Focused ion beam scanning electron microscopic (FIB-SEM) (NX2000, Hitachi High-Tech Corporation, Tokyo, Japan) images of as-spun CPI and SBS nanofibers are presented in Figure 3a, and b, respectively. To increase the uniformity of silver plating on the nanoweb surface, as-spun nanowebs should be soaked in isopropanol for at least 8 h prior to silver plating to improve their wettability in the aqueous electroless silver plating solution. However, it is insufficient for the silver-plating solution to permeate deeply into the nanofiber web because of the tiny sizes of the pores between nanofibers. Therefore, as described in the experimental section, a custom-designed silver-plating apparatus was used to improve the plating homogeneity on the nanoweb surface. This apparatus was divided into a rotating device and a silver-plating box (see SI Figure S1b). Two clips were installed in the silver-plating box to tightly fix the nanofiber web to conduct silver-plating more evenly. The silver-plating box was made of a highly hydrophobic material to prevent silver from coating the box surface during silver plating. Two essential factors affect the impedance of individual metal-plated fibers: the thickness of the plated layer and the surface-area-to-volume ratio of the metal-coated fiber [20]. The plated layer should be sufficiently thick to reduce the resistive part of the fiber. The diameter of both nanofibers significantly increases (CPI ≈ 896.5 nm, SBS ≈ 537.2 nm) after the silver-plating process relative to their as-spun nanofiber webs. Figure 3c, and d present FIB-SEM images of silver-plated CPI and SBS nanofiber webs, respectively.

### 3.2. Electrode Properties of AgNW Dry Electrodes on Agar Phantom

Ionic currents flow inside the body and are converted to electronic currents when they reach conductive electrodes to measure bioelectric potentials. The resistive and capacitive components called contact impedance are generated at this interface, where the conductive material contacts the body [6]. All the electrodes, including the Ag/AgCl electrodes, had the same contact area (approximately 63.5 mm^2^), as shown in Figure S3 (SI). Controlling and measuring the skin–electrode contact impedance is crucial when developing and designing an electrode [44]. However, contact impedance is highly resistive and capacitive, varying with the subject, location, time, and skin. Therefore, the differences in contact impedance among different types of electrodes may be highly dependent on the variation in skin impedance when a skin–electrode system is employed. Accordingly, a stable agar phantom–electrode contact system was utilized rather than a natural skin–electrode contact system in order to compare the contact impedance of each electrode used in this study, where the agar phantom is much more electrically homogeneous than human skin.

**Figure 4:**
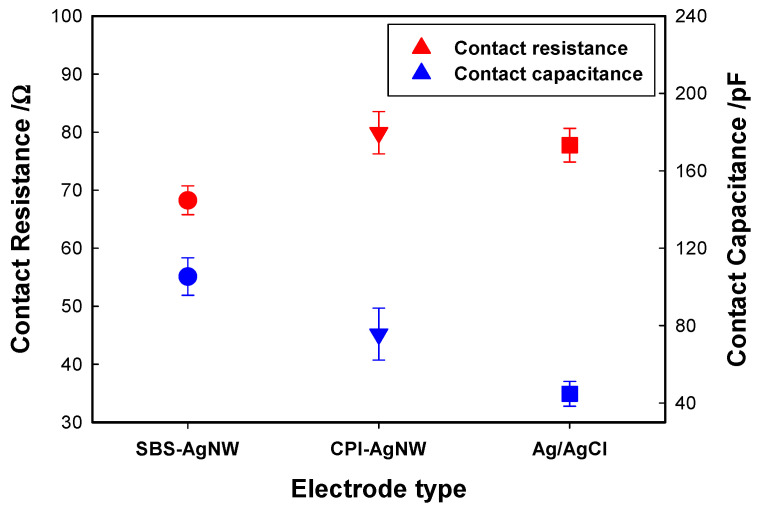
Average contact resistance and capacitance at ten frequencies ranging from 10 Hz to 500 kHz for each electrode.

Figure 4 illustrates the resistive part (R) and the capacitive part (C); the contact impedance of each electrode averaged ten frequencies, ranging from 10 Hz to 500 kHz. In terms of the resistive component of each electrode, the R values of the SBS-AgNW dry electrode were lower, and the values of the CPI-AgNW dry electrode were marginal compared to those of the metal part of the Ag/AgCl gel electrode. This result is likely the consequence of two main contributions. One is primarily due to the high electrical conductivity of AgNW dry electrodes themselves, and the other is the attribution of the contact surface area [21]. The contact area is one of the main parameters influencing contact impedance, since the nanofiber webs usually have a high specific surface area, owing to their nanoscale fiber. In contrast, the SBS-AgNW dry electrodes present comparably larger mean C values, and the CPI-AgNW dry electrode shows a slightly larger value than the Ag/AgCl gel electrode, as shown in Figure 4. In the case of the SBS-AgNW dry electrode, the capacitors created between many conductive fibers are accumulated to provide a larger capacitance, but the electrical resistance is smaller through many contact points than in the CPI-AgNW dry electrode. For the capacitive (C) part, a higher value of C results in a lower contact impedance when increasing the frequency of the injected AC source.

A significant issue considering nanofiber web silver plating is that the metal-coated fiber structure usually has large capacitive properties, which may cause signal distortion [20]. In addition, the capacitance created by the gap between fibers and the contact resistance caused by fiber-to-fiber contacts plays an essential role in the impedance of rubber–AgNW dry electrodes. In general, nanofiber webs are highly porous, and air occupies about 75% of the volume of the web. Due to the increased diameter of nanofibers after the silver-plating process, the gap between fibers can be diminished to a certain degree to further decrease the capacitive part of the rubber–AgNW dry electrode. Moreover, one satisfying result obtained from an improved silver-plating process is that most fibers are strongly connected by reduced silver, as shown in Figure 3c,d, which can further reduce fiber–fiber contact resistance, contributing to a decrease in the total impedance of AgNW dry electrodes.

**Figure 5:**
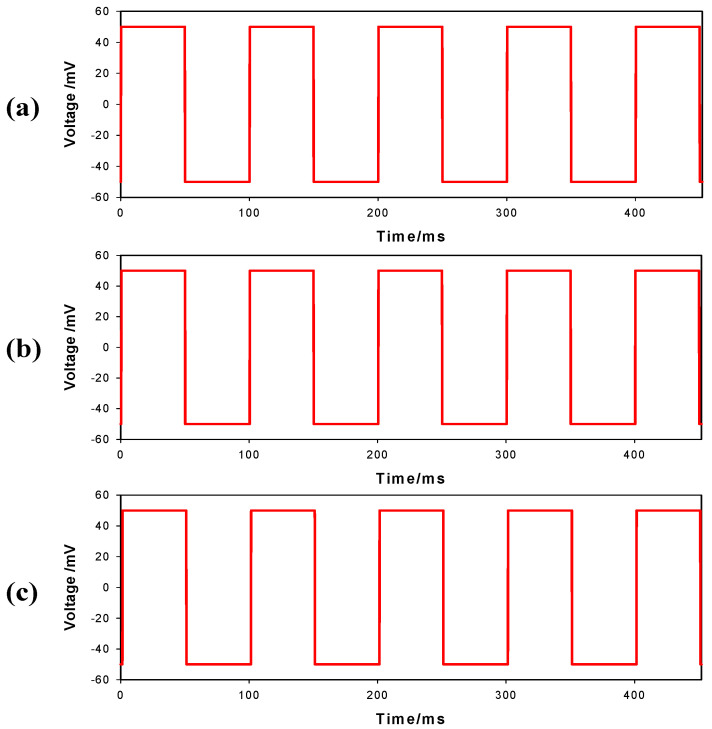
Step response of SBS-AgNW dry electrode (**a**), CPI-AgNW dry electrode (**b**), and Ag/AgCl gel electrode (**c**) when injecting square waveforms of 10 mA current with a 100 ms period and 50% duty cycle.

Figure 5 represents the step-response voltage waveforms of the SBS-AgNW dry electrode (a), CPI-AgNW dry electrode (b), and Ag/AgCl gel electrode (c) without signal distortion when injecting square waveforms of 10 mA current with a 100 ms period and 50% duty cycle imposed on the compact structure of the AgNW dry electrodes, which leads to less signal distortion. Unlike our results, a textile fabric-based electrode exhibited a noticeably distorted signal during the step-response test due to the porous structure affecting the capacitive part of the electrodes and causing signal distortion [4]. When comparing these results, we expected satisfactory performance of the proposed AgNW dry electrodes with little distortion in the measurement of bioelectric potentials with components in a wide frequency range or in measuring electrical properties using exogenic high-frequency components.

**Figure 6:**
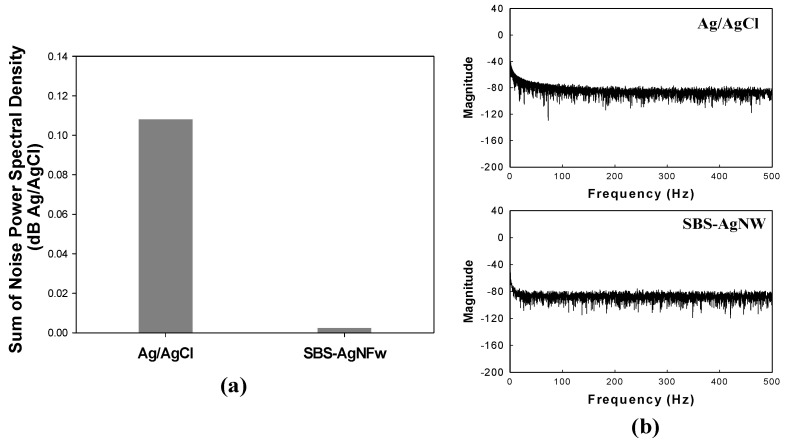
The sum of noise power spectral density of each electrode (**a**) and all values normalized to correct for overall electrode impedances in each electrode (**b**).

When evaluating new electrodes, the noise characteristic is an inevitable parameter. Specifically, the given low amplitude signal of bioelectric potentials is less than a few tens of mV in magnitude. Though many factors affect the noise characteristic of the electrode, its contact impedance largely governs the noise level. Each sum of noise power spectral density of Ag/AgCl and SBS-AgNW is illustrated in Figure 6a, and all values were normalized to the gain of Ag/AgCl electrodes to correct for the differences in gain of the SBS-AgNW electrodes, as presented in Figure 6b. The SBS nanoweb dry electrode detects much less noise than the Ag/AgCl gel electrode.

### 3.3. Biopotential Recording

Human volunteer tests were carried out to compare the realistic performance of rubber-AgNW dry electrodes with that of clinically used Ag/AgCl gel electrodes. Since the above-evaluated results of the agar phantom–electrode contact system, which was examined for an AgNW dry electrode via comparisons with Ag/AgCl gel electrodes, are insufficient to estimate actual performance in a realistic environment, a stable agar phantom cannot reflect the natural dynamics and complicated conditions encountered on human skin. Furthermore, to assess our system, in this study, CPI and SBS (both rubber-AgNW dry electrodes) were selected to perform the subject test using ECG and EMG measurements.

Bioelectric potentials are generated due to changes in distributed ions in the body, and these potentials can be measured by electrodes when they are transferred to the body surface [21]. The currents should pass through the skin in this process, which has a high impedance compared to the human body’s conductivity, mainly due to the stratum corneum layer of the skin, a top layer of skin consisting of many sublayers of compacted, flattened, non-nucleated dehydrated corneocytes filled with cross-linked keratin [1]. The stratum corneum may have a thickness of about 10–40 µm and typically has around a few hundred kΩ to one MΩ per square centimeter for a 10 Hz input signal frequency [44,45,46]. Therefore, this layer significantly inhibits the transfer of electric signals from the tissue to the electrode [5,20,46].

**Figure 7:**
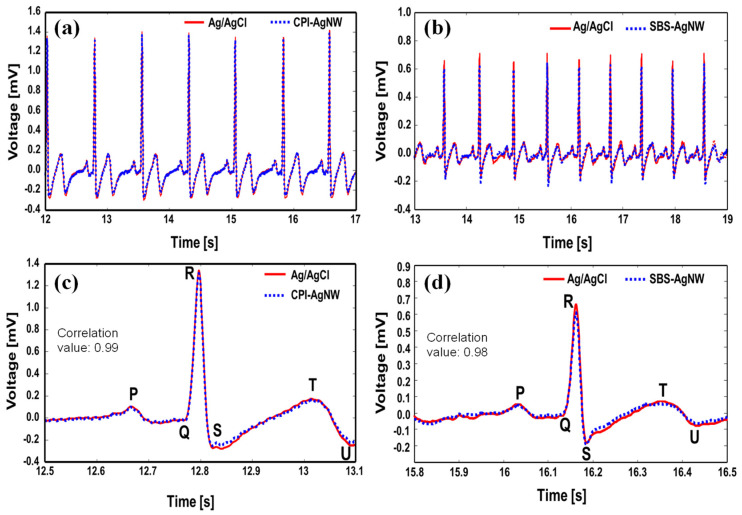
ECG recordings of the CPI-AgNW dry electrode and Ag/AgCl gel electrode (**a**); SBS-AgNW dry electrode and Ag/AgCl gel electrode (**b**); a comparison of the P, Q, R, S, T, and U features of ECG waveforms between the CPI-AgNW dry electrode and Ag/AgCl gel electrode (**c**); and SBS-AgNW dry electrode and Ag/AgCl gel electrode (**d**).

Electrocardiography (ECG) is the primary diagnostic signal in patients with cardiac diseases and interprets the heart’s electrical activity at a given time [6,47]. Since the extracted features from ECG waveforms and repeated patterns can provide meaningful information about a subject’s heart and hemodynamic status, it is important to measure ECG waveforms without distortion and deformation. Hence, a clinically acceptable, high-quality ECG signal can only be used to diagnose cardiac diseases and is a standard procedure in current cardiac treatment [6,20,47,48]. Therefore, ECG detected from any alternative electrode, i.e., the rubber-AgNW dry electrode, should be as accurate as that detected from the standard and clinically verified Ag/AgCl gel electrode. EEG signals simultaneously recorded using CPI-AgNW dry electrodes and Ag/AgCl gel electrodes (a); SBS-AgNW dry electrodes and Ag/AgCl gel electrodes (b); a comparison of P, Q, R, S, T, and U features of ECG waveforms between the Ag/AgCl gel electrode and the CPI-AgNW dry electrode (c); and Ag/AgCl gel electrodes and SBS-AgNW dry electrodes (d) are presented in Figure 7. The correlation between the rubber-AgNW dry electrode and Ag/AgCl gel electrode was strictly observed, and correlation coefficients of 0.99 and 0.98 were found between the CPI-AgNW dry electrode–Ag/AgCl gel electrode and the SBS-AgNW dry electrode–Ag/AgCl gel electrode, respectively, which indicates that they achieve almost the same level of performance in ECG measurement. However, negligibly less or more signal intensity can be observed, particularly between the SBS-AgNW and Ag/AgCl electrode cases, as shown in Figure 7b, due to the differences in electrode positions of the two electrode pairs.

Electromyography (EMG) is a clinically well-known biopotential for evaluating and recording the electrical activity produced by skeletal muscles. It is used in various applications, including diagnosis of neuromuscular disorders, monitoring of muscular fatigue, investigation of muscle function, and as a control input for prosthetic devices [49]. However, electrodes used to measure EMG were quite challenging because EMG signals had a broader bandwidth of 10 Hz to 500 Hz. Moreover, the positions of the electrodes are also important during recording to obtain an accurate and equivalent EMG signal for both electrodes. The electrodes should be placed in an appropriate location, as presented in Figure 1b,c, to obtain a better correlation value for the EMG signals from the electrode pair. In this study, EMG signals were diagnosed for hand and leg muscle activities.

Rubber-AgNW dry electrodes and Ag/AgCl gel reference electrodes were used to simultaneously record the EMG signals. The raw EMG data recorded for hand muscle activities corresponds to a correlation coefficient between the CPI-AgNW dry electrode and the Ag/AgCl gel electrode of 0.69 and between the SBS-AgNW dry electrode and the Ag/AgCl gel electrode of 0.99. The SBS-AgNW dry electrode shows a better correlation than the CPI-AgNW dry electrode with the Ag/AgCl gel electrode. The SBS-AgNW dry electrode achieves almost the same performance in concurrently acquiring signals as Ag/AgCl electrodes.

**Figure 8:**
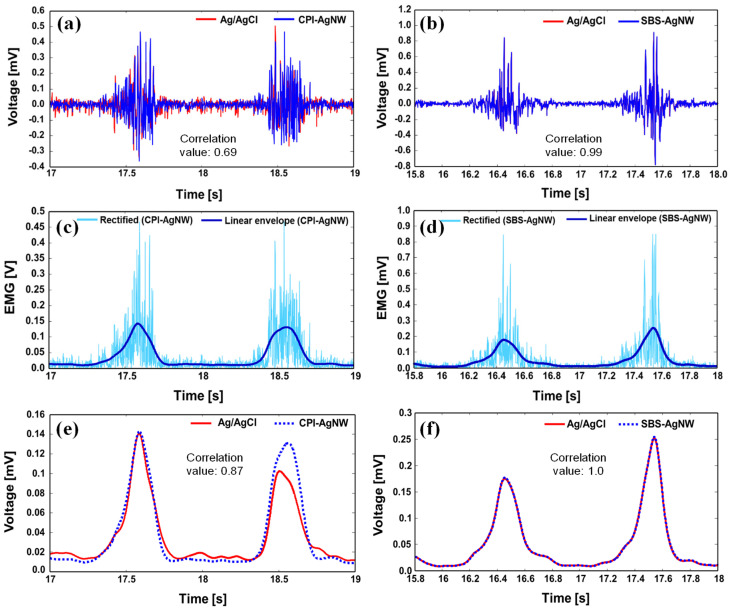
A comparison of raw EMG data recorded from hand muscle activities using a CPI-AgNW dry electrode and Ag/AgCl gel electrode (**a**), SBS-AgNW dry electrode and Ag/AgCl gel electrode (**b**), a rectified envelope of raw EMG signal collected for the CPI-AgNW dry electrode (**c**), SBS-AgNW dry electrode (**d**), and a comparison of the envelope of EMG signals for the CPI-AgNW dry electrode and Ag/AgCl gel electrode (**e**) and for the SBS-AgNW dry electrode and the Ag/AgCl gel electrode (**f**).

Since post-processed EMG data are used in many applications, we further rectified and digitally low-pass-filtered the raw EMG signals using a fifth-order Butterworth filter with a cutoff frequency of 5 Hz [50]. After applying this filtering process, the correlation coefficient between the CPI-AgNW dry electrode and the Ag/AgCl gel electrode increased to 0.87. Interestingly, the correlation coefficient between the SBS-AgNW dry electrode and the Ag/AgCl gel electrode, on the other hand, increased to 1.00. Overall, the SBS-AgNW dry electrode shows a better correlation than the CPI-AgNW dry electrode with the Ag/AgCl gel electrode in the simultaneous acquisition of signals. This indicates that they offer a comparable performance level in EMG recording for hand muscle activities. Figure 8 represents a comparison of raw EMG data recorded for hand muscle activities using a CPI-AgNW dry electrode with the Ag/AgCl gel electrode and SBS-AgNW dry electrode with Ag/AgCl gel electrode ((a) and (b), respectively). A rectified envelope of raw EMG signal was collected for the CPI-AgNW dry electrode and SBS-AgNW dry electrode ((c) and (d), respectively). A comparison of the envelope of EMG signals for the CPI-AgNW dry electrode with the Ag/AgCl gel electrode and the SBS-AgNW dry with the Ag/AgCl gel electrode is shown in (e) and (f), respectively.

**Figure 9:**
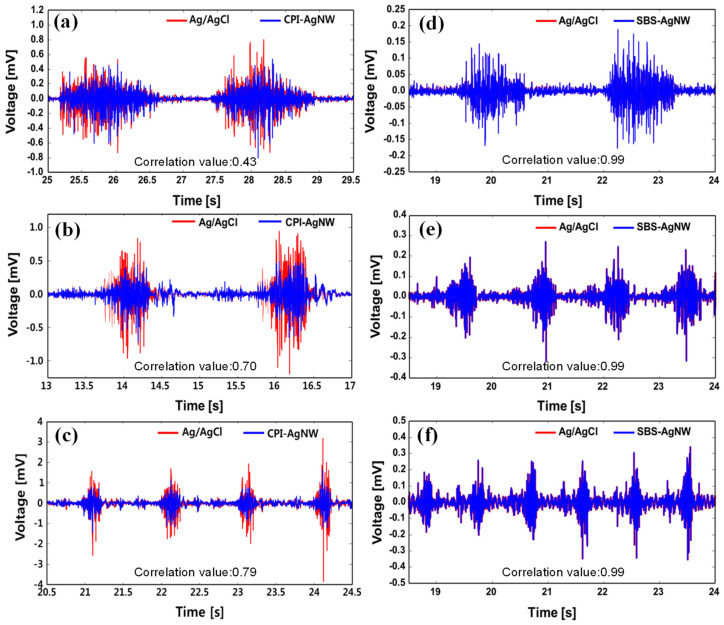
A comparison of raw EMG data recorded from leg muscles during various activities, such as no walking but clenching and expanding of leg fingers (**a**), walking at 3 km/h (**b**), and walking at 6 km/h (**c**) on treadmill using the Ag/AgCl gel electrode and CPI-AgNW dry electrode. (**d**–**f**) for Ag/AgCl gel electrode and SBS-AgNW dry electrode at no walking, walking at 3 km/h, and walking at 6 km/h, respectively.

EMG recorded raw signals of leg muscle activities, such as a standing position (not walking, but the subject was asked to slightly clench and unclench his foot soles repeatedly during the test) and walking at speeds of 3 km/h and 6 km/h on a treadmill. The correlation coefficient between the CPI-AgNW dry electrode and Ag/AgCl gel electrode is 0.43, 0.70, and 0.79, respectively, as observed in Figure 9a–c. Similarly, Figure 9d–f represents a comparison of raw EMG data recorded in leg muscles in a standing position and walking at speeds of 3 km/h and 6 km/h speed using an SBS-AgNW dry electrode and Ag/AgCl gel electrode. The raw EMG signals were recorded for various leg muscle activities, i.e., at standing conditions, walking at speeds of 3 km/h and 6 km/h. The correlation coefficient between the SBS-AgNW dry electrode and Ag/AgCl gel electrode shows the same value of 0.99. Interestingly, the EMG signals of the SBS-AgNW dry electrode and Ag/AgCl gel electrode EMG signals are almost overlapped. There is no distinction of correlation in various muscle activities such as standing position and walking at speeds of 3 km/h and 6 km/h. On the other hand, the raw EMG signals from the CPI-AgNW dry electrode exhibited lower intensity than those from the Ag/AgCl gel electrode. Therefore, it can be concluded that the SBS-AgNW dry electrode shows noticeably better performance than the CPI-AgNW dry electrode in terms of EMG recording of leg muscles performing various activities, which is almost the same level of performance as the clinically used Ag/AgCl gel electrode. After all, for both the CPI-AgNW, SBS-AgNW dry electrode, and Ag/AgCl gel electrode, it is observed that the leg muscle activities were also identified equidistantly with the increased walking speed is visible in signaling frequency as per leg muscles activities.

### 3.4. EIT Test with Multiple Electrodes

The fundamental principle behind EIT is to non-invasively detect impedance variations within the object/body by repeatedly recording surface potentials arising from the rotating injection of a known small, high-frequency AC current through electrodes adhered to the object’s circumference. Then, the measurements are reconstructed to form images using a specific algorithm [9,43,51]. Recently, EIT was applied to monitor the regional lung function of an intensive care patient to prevent ventilator-induced lung injury by guiding positive end-expiratory pressure (PEEP) trials [51,52,53]. To produce a stable output of images, dry electrodes that do not change their characteristics over time and have a small variation in characteristics among electrodes are required since the use of many wet electrodes causes skin irritation and deterioration in performance over time. Based on the previous excellent biopotential recordings at a higher frequency band in agar phantoms, an EIT test was conducted with the SBS-AgNW dry electrode and the Ag/AgCl gel electrode for comparison.

**Figure 10:**
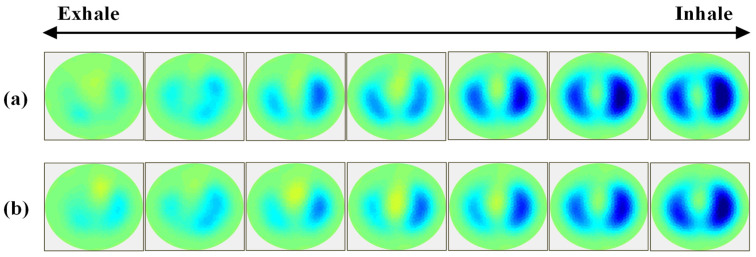
Series of time-difference pulmonary images representing air volume changes during inspiration recorded through the EIT system at an injection current of 1 mA and a frequency of 10 kHz using Ag/AgCl gel electrodes (**a**) and SBS-AgNW dry electrodes (**b**). The yellow, green, and blue denote high, medium, and low impedance, respectively.

A series of time-difference impedance images representing air volume changes during inspiration and expiration was recorded through the EIT system at an injection current of 1 mA and a frequency of 10 kHz using Ag/AgCl gel electrodes and SBS-AgNW dry electrodes, as illustrated in Figure 10a, and b, respectively. The reconstructed image corresponding to the end of exhalation was set as a reference, as shown in the leftmost image of each line in Figure 10; the rightmost image corresponds to the end of inhalation. Each series of time-difference lung images presents step-by-step gradual respiration. The SBS-AgNW dry electrode shows a lung EIT image similar to that of the Ag/AgCl gel electrode. However, a very negligible difference can be seen in the two series of lung images, since were not recorded simultaneously. Therefore, each image cannot represent the same time situation. In addition, the EIT image is affected by the three-dimensional lung structure and blood diffusion in a subject, which may vary considerably depending on the location of the attached electrodes. In the case of the Ag/AgCl electrode, the contact area of the gel is larger than that of the SBS-AgNW dry electrode. No matter how fairly they are tested, a specific difference in the two series of lung images may be induced. Hence, the reconstructed images do not precisely represent the actual cross-sectional lung where the electrodes were placed. On the other hand, the subject’s chest cross-section is not a perfectly circular shape, so the reconstructed lung images may also be deformed when a circular domain is employed in an image reconstruction algorithm. Overall, the SBS-AgNW dry electrode shows comparable performance in lung EIT image reconstruction to that of the medically verified Ag/AgCl electrode.

### 3.5. Electrode Stability Test

Developments in ubiquitous healthcare systems require long-term biopotential monitoring of patients [4]. Clinically verified Ag/AgCl gel electrodes cannot be applied in long-term applications because of their performance deterioration over time as their conductive gel pads dry [4,5,18,19,20,21]. Therefore, electrode stability may be a significant parameter in evaluating electrode performance in long-term settings. For a detailed assessment of electrode stability see Supplementary Information, S3.

**Figure 11:**
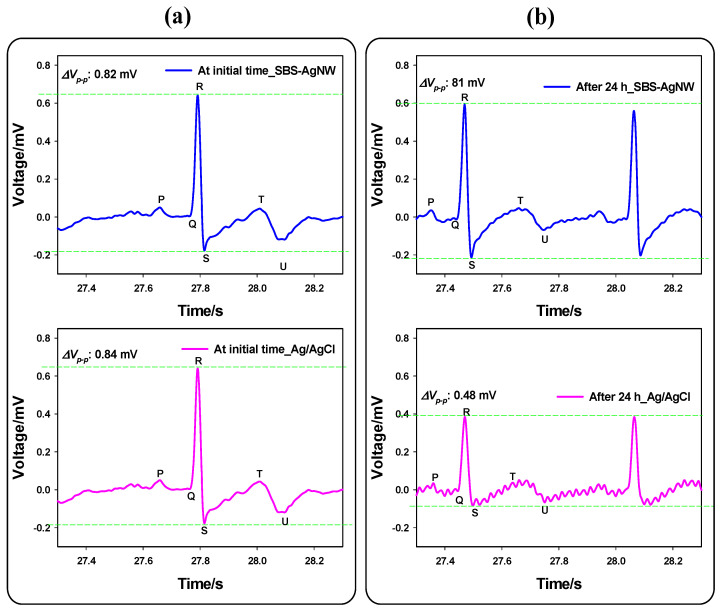
A single ECG signal of the SBS-AgNW dry electrode (blue) and Ag/AgCl gel electrode (pink) upon initial recording (**a**) and after 24 h continuously attached to the subjects’ chest (**b**) for the electrode stability test. The green dot line indicates the peak-to-peak voltage range in the y-axis scale.

The initially recorded ECG signals from the SBS-AgNW dry electrode and Ag/AgCl gel electrode show no remarkable variation in signal intensity. The P, Q, R, S, T, and U features of the obtained signal on the ECG waveform are almost identical for the two electrode cases, as depicted in Figure 11a. On the other hand, the ECG signal recorded from the Ag/AgCl gel electrode after 24 h offers more noisy and significantly degraded intensity, along with somewhat distorted P, S, and T waves, whereas the signal collected concurrently from the SBS-AgNW dry electrode after 24 h exhibits a nearly unaltered intensity compared to its initial recorded signal, as depicted in Figure 11b. It is also observed that the recorded heartbeat rate after 24 h is quicker than that at the initial signal in both electrode scenarios, most likely due to physical stress caused by the subject’s continuous wearing of electrodes for 24 h throughout the experiment without sufficient sleep and frequent breathing.

**Figure 12:**
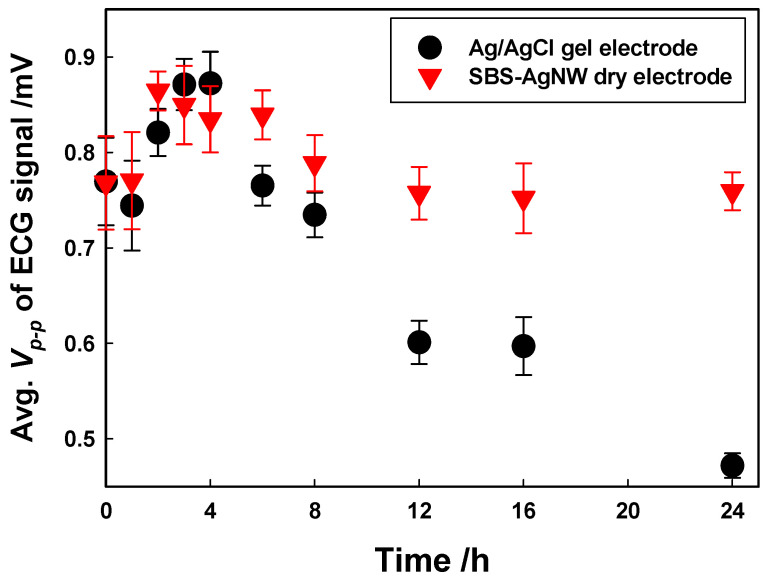
The average peak-to-peak voltage (V_p-p_) intensity of the ECG signal was obtained for SBS-AgNW and Ag/AgCl electrodes at various time intervals until 24 h.

Figure 12 depicts the peak-to-peak voltage intensity of the obtained ECG signal for the SBS-AgNW and Ag/AgCl electrodes at various time intervals. Since all the obtained ECG voltage intensity was not uniform in each time interval, we considered the average amplitude of R peaks from 25 ECG pulses for both electrodes to fairly evaluate the signal intensity. As an example, 25 continuous ECG pulses and their averaged voltage signals recorded at the beginning and obtained after 24 h, respectively are roughly illustrated in Figure S6a, and b (SI). Figure 12 shows that the recorded ECG signal intensities were relatively equivalent for both the SBS-AgNW and Ag/AgCl gel electrodes for ~8 h of constantly being attached to the subject’s chest; however, successive recorded signals for Ag/AgCl gel electrodes steadily deteriorated after 8 h. In comparison, the SBS-AgNW dry electrode displayed practically the same signal intensities until 24 h recording. If this experiment could be prolonged for more than 24 h, distinct signal intensities between the SBS-AgNW dry electrode and the Ag/AgCl gel electrode could possibly be detected. In the case of the Ag/AgCl gel electrode, the gel part usually contacts the skin surface, and the gel in the conductive pad gradually dries due to its long-term use. Hence, the resistive part of contact impedance is steadily increased, degrading electrode performance [6,7]. The SBS-AgNW dry electrode, on the other hand, is devoid of gel or adhesiveness and is affixed to the subject’s chest using additional Scotch tape. The state of electrode–skin surface contact was sustained for a longer duration due to the nanoweb surface characteristics and dry electrode fabrication technique described in the “Electrode Properties of AgNW Dry Electrodes on Agar Phantom” section. Thus, SBS-AgNW dry electrodes can be applied in long-term applications for biopotential recording in human subjects without deteriorating electrode performance. Moreover, the same AgNW dry electrode can be used several times for monitoring, while an Ag/AgCl gel electrode is only usable once after opening its gel pad. However, the long-term stability and performance of Ag/AgCl gel electrodes may vary from subject to subject due to disruption of skin–electrode contact resulting from excessive facial hair or, to a lesser extent, body sweat tendencies, which may diminish skin–electrode contact when the electrode gel pad is attached to the skin for an extended period of time.

## 4. Conclusions

CPI and SBS-AgNW dry electrodes were successfully fabricated in this work through electrospinning and silver-plating processes. The key electrode properties of agar phantoms, such as contact impedance, step response, and noise characteristics (for SBS-AgNW dry electrode), as well as biopotential recordings, such as ECG and EMG recording for hand and leg muscle activities for both the SBS-AgNW dry electrode CPI-AgNW dry electrodes, were investigated and compared with Ag/AgCl gel electrodes. Subsequently, the SBS-AgNW dry electrode was comprehensively examined in terms of respiratory monitoring of EIT using multiple electrodes. The results indicate that the SBS-AgNW dry electrode was identical to the verified Ag/AgCl gel electrode in terms of performance. Furthermore, the SBS-AgNW dry electrode was recognized to be more beneficial than the Ag/AgCl gel electrode through EIT respiratory monitoring using multiple electrodes for high-frequency signals and ECG recordings for long-term stability. Therefore, to quantitatively evaluate rubber-based AgNW dry electrodes, a simple eyelet-shaped electrode type was selected; however, other applications are also available, since rubber-AgNWs are thin, flexible, washable, and reusable and can be directly integrated into wearable devices and electric circuits due to their size. This can enable continuous monitoring of the subject’s EMG, ECG/pulse rate, lung functioning and wirelessly transmit the results to personal electronics is a highly desired approach to realize dynamic and real-time health care and prevent cardiovascular disease, and identify physical anomalies in daily life.

## 5. Patents

Hongdoo Kim, Kap Jin Kim, Mohammad Shamim Reza, M. K. Yoo, Method for manufacturing dry electrode based on elastic nanofiber web, Patent Office: KR, Patent Number KR101947895B1, https://patents.google.com/patent/KR101947895B1/en?oq=10-1947895, (accessed on 13 February 2019).

## Data Availability

The data presented in this study are available upon request from the corresponding author.

## Supplementary Materials

The following supporting information can be downloaded at: https://www.mdpi.com/article/10.3390/s23177377/s1, Provided as a separate file containing methodology for fabrication of nanofiber web-based dry electrode: (S1) Methods for three-electrode setup and four-electrode setup on the agar phantom to characterize the properties of the electrode, i.e., contact impedance, step response, and noise characteristics; (S2) Electrode stability test procedure; (S3) Experimental methodology, electrode characterization, and stability test results, with additional figures (Figures S1–S6) [8,30,31,46,54,55].
