# Supplementary material for: Electrospun Rubber Nanofiber Web-Based Dry Electrodes for Biopotential Monitoring
Source: Sensors (Basel). 2023 Aug 24;23(17):7377. doi: 10.3390/s23177377 (PMC10490276; doi:10.3390/s23177377)
Supplement: Supplementary file 1 [file sensors-23-07377-s001.zip › sensors-2429288-supplementary.pdf]

Supporting Information

Electrospun Rubber Nanofiber Web-Based Dry Electrodes for Biopotential Monitoring

Mohammad Shamim Reza [1], Lu Jin [1,†], You Jeong Jeong [2], Tong In Oh [2], Hongdoo Kim [1,*] and Kap Jin Kim [1,*]

[1] Department of Advanced Materials Engineering for Information & Electronics, Kyung Hee University, Gyeonggi-do, Yongin 17104, Republic of Korea; shamimtex2012@gmail.com (M.S.R.); jinlu1011@hotmail.com (L.J.)
[2] Department of Biomedical Engineering, School of Medicine, Kyung Hee University, Seoul 02447, Republic of Korea; yj.jeong0107@gmail.com (Y.J.J.); tioh@khu.ac.kr (T.I.O.)
* Correspondence: hdkim@khu.ac.kr (H.K.); kjkim@khu.ac.kr (K.J.K.)
† Current address: Yiwu Research Institute of Fudan University, Yiwu 322000, China.

Supplementary Information

S1. Methodology

During electrospinning, a positive DC voltage of 22 kV was applied to the SBS solution at a flow rate of 2.5 ml/h, 15~16 cm of TCD (Tip to collector distance), and a 23 G needle was used. In the case of CPI solution, the DC voltage was set at 14 kV, and a flow rate of 1.2 ml/h, 12 cm of TCD, and a similar needle were fitted in the syringe at the fiber collector cylinder rotation speed of 60 RPM. A conductive Teflon-coated PET sheet was used to collect the electrospun nanofiber web. The purpose of adding THF to the CPI electrospun solution is to increase the volatility of the solvents during the electrospinning process.

The silver-plating technique was adapted to the SBS and CPI electrospun nanofiber web plating, like the basic principle of the electroless silver plating technique. However, to improve the uniformity and electrical conductivity of the Ag-nanofiber web, the concentration of each plating solution was modified to slow the reaction rate as follows; at first, three different solutions were prepared: a 0.1 M solution of silver nitrate, a 0.8 M solution of sodium hydroxide (Samchun Chemical Co. Ltd, Korea) and a 0.25 M solution of dextrose (Duksan Co., Korea). Then, the silver nitrate solution was mixed with the sodium hydroxide solution at the ratio of 2/1 (v/v) and stirred until becoming brown precipitation. The ammonia water /28~30% (Samchun Chemical Co. Ltd, Korea) was added dropwise until it dissolved to form a water-soluble diamine silver complex compound. After that, the diamine silver complex clear solution was poured into a custom-designed silver-plating box where Ag-NPs containing nanofiber webs were pre-installed, as shown in **Figure S1 (b).** Then, the dextrose solution was added into the silver-plating box at the ultimate ratio, 10/5/1 (v/v/v) of silver nitrate, sodium hydroxide, and dextrose solutions. The silver-plating equipment box was rotated at 10 rpm for 45 min to obtain even silver plating. During the plating,

the diamine silver complex cation ($[Ag(NH_3)_2]^+$) was reduced gradually by dextrose to silver, which was deposited preferentially on the nanofiber web surface. It is noted that as-spun nanowebs should be immersed into 2-propanol for at least 8 h before silver plating to further enhance their wettability unto the aqueous electroless silver plating solution, which is helpful to provide uniform silver plating from the surface to deep inside of the nanofiber web. **Figure S1** illustrates a manufacturing process of rubber-AgNW dry electrodes: schematic of electrospinning set-up to fabricate silver nanoparticles embedded nanofiber web using deep-brown silver nanoparticles enriched electrospun rubber solution **(a)**, photographs of custom-designed silver-plating equipment set-up **(b)**, a microscopic image of silver-plated nanofiber web **(c)**, photographs of SBS-AgNW and CPI-AgNW dry electrode **(d)**. **Figure S2** illustrates photographs of contact angle measurements of a water drop on the surface of as-electrospun CPI **(a)**, SBS **(b)** nanofiber web, and the CPI **(c)** and SBS **(d)** nanofiber webs after an 8-h dip in isopropanol. The water contact angle of isopropanol-dipped nanowebs decreased to half that of as-spun nanofiber webs. As a result, isopropanol-dipping greatly improves the hydrophilicity of the nanoweb.

A diameter of 9 mm was determined as the size of the AgNW dry electrode, where the shape of the electrodes was strictly maintained, which is the same as the metal part size of the Ag/AgCl gel electrode (Red Dot 2223, 3M, USA). A metal eyelet structure was adopted for the fabricated AgNW dry electrodes. A thick polyester mesh fabric piece was placed between the metal eyelet and nanoweb to provide the AgNW dry electrode with appropriate elasticity and softness, which provided tight skin-electrode contact. A cross-sectional schematic of the fabricated nanoweb-based dry electrode and Ag/AgCl gel electrode **(a)** and the placing order of both the electrodes on human subjects for biopotential recording **(b)** is illustrated in **Figure S3**.

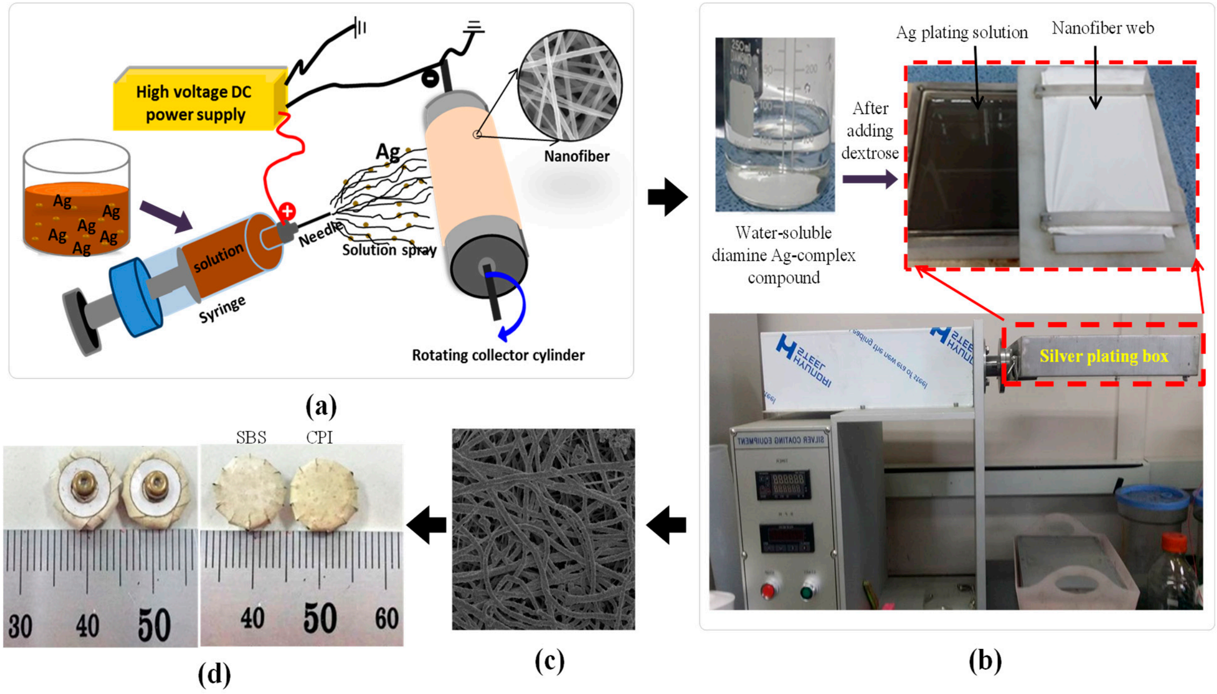

Figure S1. The manufacturing process of rubber-AgNW dry electrodes: schematic of electrospinning set-up to fabricate silver nanoparticles embedded nanofiber web using deep-brown silver nanoparticles enriched electrospun rubber solution **(a)**, photographs of custom-designed silver-plating equipment set-up **(b)**, a microscopic image of silver-plated nanofiber web **(c)**, photographs of SBS-AgNW and CPI-AgNW dry electrode **(d)**.

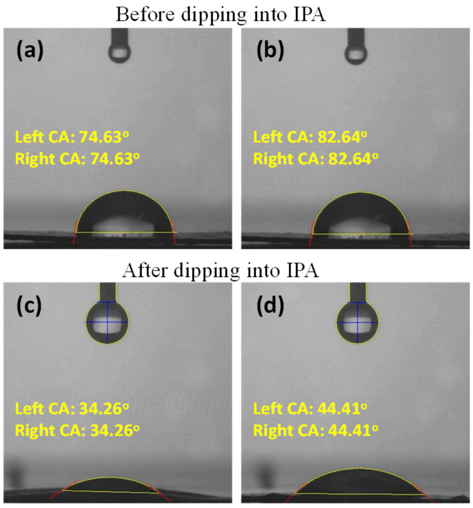

Figure S2. Contact angle measurements of a water drop on the surface of as-electrospun CPI **(a)**, SBS **(b)** nanofiber web, and the CPI **(c)** and SBS **(d)** nanofiber web after dipping into the isopropanol for 8h.

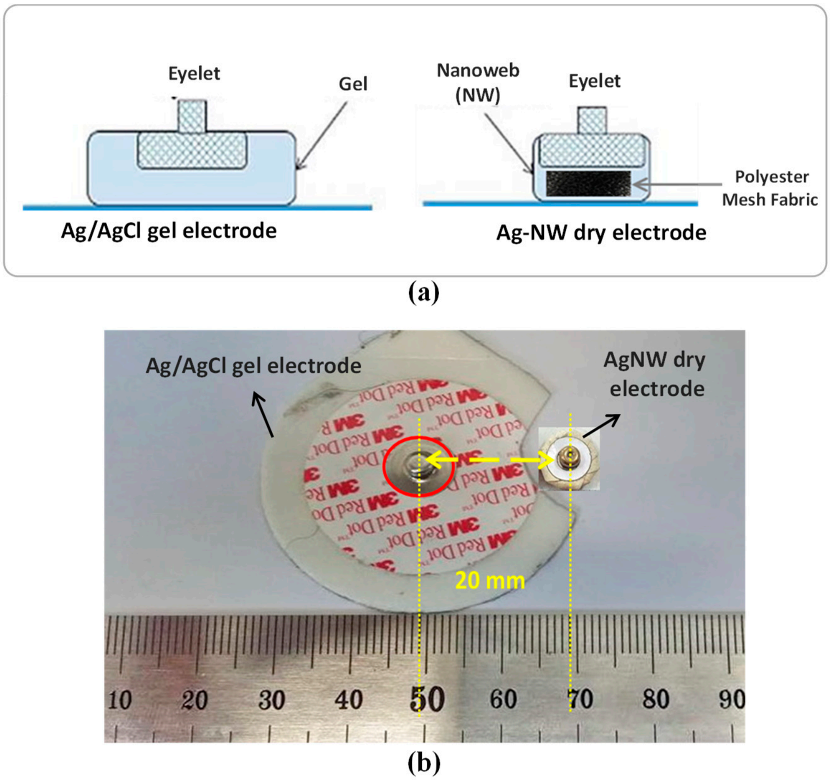

Figure S3. A cross-sectional schematic of a fabricated nanoweb-based dry electrode and Ag/AgCl gel electrode **(a)**, Ag/AgCl gel electrode (Red Dot 2223, 3M, USA), and AgNW dry electrode placing order on a human subject for biopotential recording **(b)**.

S2. Characterization

The electrode-electrolyte contact impedance should be stable and as low as possible to minimize the loading effect and common-mode noise at the amplifier input stage [1,2]. The impedance analyzer (1260A, AMETEK Inc., Pennsylvania, USA) was employed to measure the contact impedances of the electrodes at frequencies of 10, 50, 100, 1k, 5k, 10k, 50k, 100k, 250k, and 500k were selected [3]. As shown in **Figure S4(a)**, a three-electrode setup was used to measure the impedance, Z_3, which is equivalent to the sum of the agar impedance and electrode contact impedance at each selected frequency [4]. Likewise, a four-electrode setup was employed to measure the impedance, Z_4, equivalent to the agar impedance, at each selected frequency, as shown in **Figure S4(b)** [5]. Consequently, the electrode impedance along with the electrode-agar surface impedance can be easily calculated by subtracting the measured four-electrode impedance from the three-electrode value at each frequency, i.e., $Z_M = Z_3 - Z_4$. The measured contact impedance, Z_M, of each electrode on the agar phantom can be modeled as a parallel RC circuit, which is expressed by the following equation:

$$Z_M = R_M + iX_M = \frac{R}{1 + (2\pi fRC)^2} - i\,\frac{2\pi fR^2C}{1 + (2\pi fRC)^2} \qquad (i)$$

Here, the equivalent resistance and capacitive reactance of the electrode are denoted by R_M and X_M, respectively. These were measured using the impedance analyzer, and f is the frequency of the applying voltage. Thus, the actual R and C values for each electrode were obtained by rearranging the equation (i) as follows:

$$R = \frac{R_M^2 + X_M^2}{R_M}, \qquad C = \frac{-X_M}{2\pi f(R_M^2 + X_M^2)} \qquad (ii)$$

Since the values of *R* and *C* were as a function of frequency, the *R* and *C* values were averaged over the selected frequencies using a least-squares algorithm to compare each type of electrode more accurately [6].

The step response characteristics of each electrode were evaluated by utilizing the four-electrode measurement setup with an agar phantom. Between the outer pair of electrodes, we injected 10 mA current pulses with 10 ms duration and 50% duty cycle. Simultaneously, using a data acquisition system, MP36 (Biopac Systems, USA), voltage pulses between the inner pair of electrodes were measured at a 1 kHz sampling frequency.

Likewise, the voltage between the inner pair of electrodes was measured without injecting current using the same setup for step response measurement. Any non-zero voltage measurement was considered noise. The sum of each electrode's noise power spectral density was analyzed and compared.

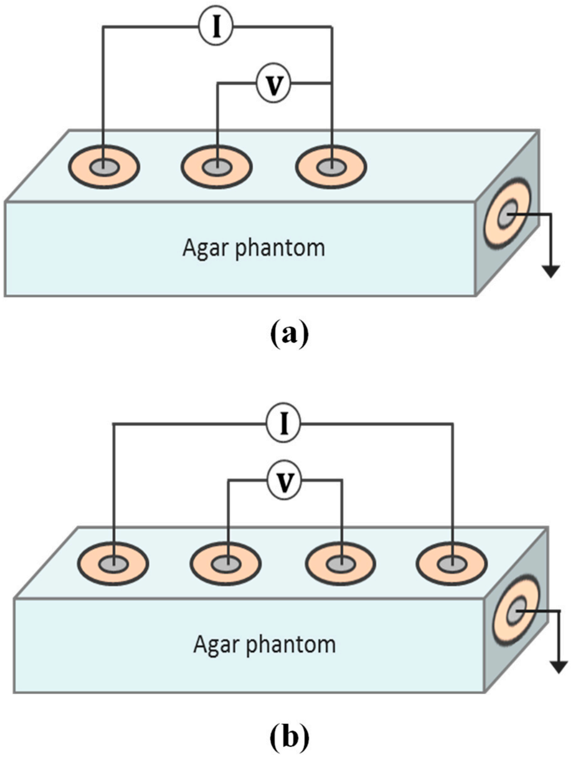

Figure S4. Schematics of an experimental setup for three-electrode impedance measurement **(a)**, and four-electrode impedance measurement **(b)** to obtain the contact impedance of each electrode [6].

S3. Electrode Stability Test

Electrode stability tests were subjected to the human body in terms of ECG monitoring. For this particular study, the physical information of the human subject was as follows; sex: male, height: 165.5 cm; weight: 65 kg; age: 28 years; race: Indian. For ECG monitoring, both the SBS-AgNW dry electrode and, as a reference, an Ag/AgCl gel electrode (Red Dot 2223, 3M, USA) were continuously attached to subjects' chests in the desired positions for 24 h without shifting or detaching any electrode from their initial positions. ECG signal was then simultaneously recorded for both the SBS-AgNW dry electrode and Ag/AgCl gel electrode under specific time intervals

until 24 h, such as at the initial time after attaching the electrode to subjects' chests, after 1h, 2 h, 3 h, 4 h, 6 h, 8 h, 12 h, 16 h, and finally at 24 h, as presented the screenshot image of ECG recording in **Figure S5**. The obtained signals from the SBS-AgNW dry electrode and Ag/AgCl gel electrode are blue and pink, respectively.

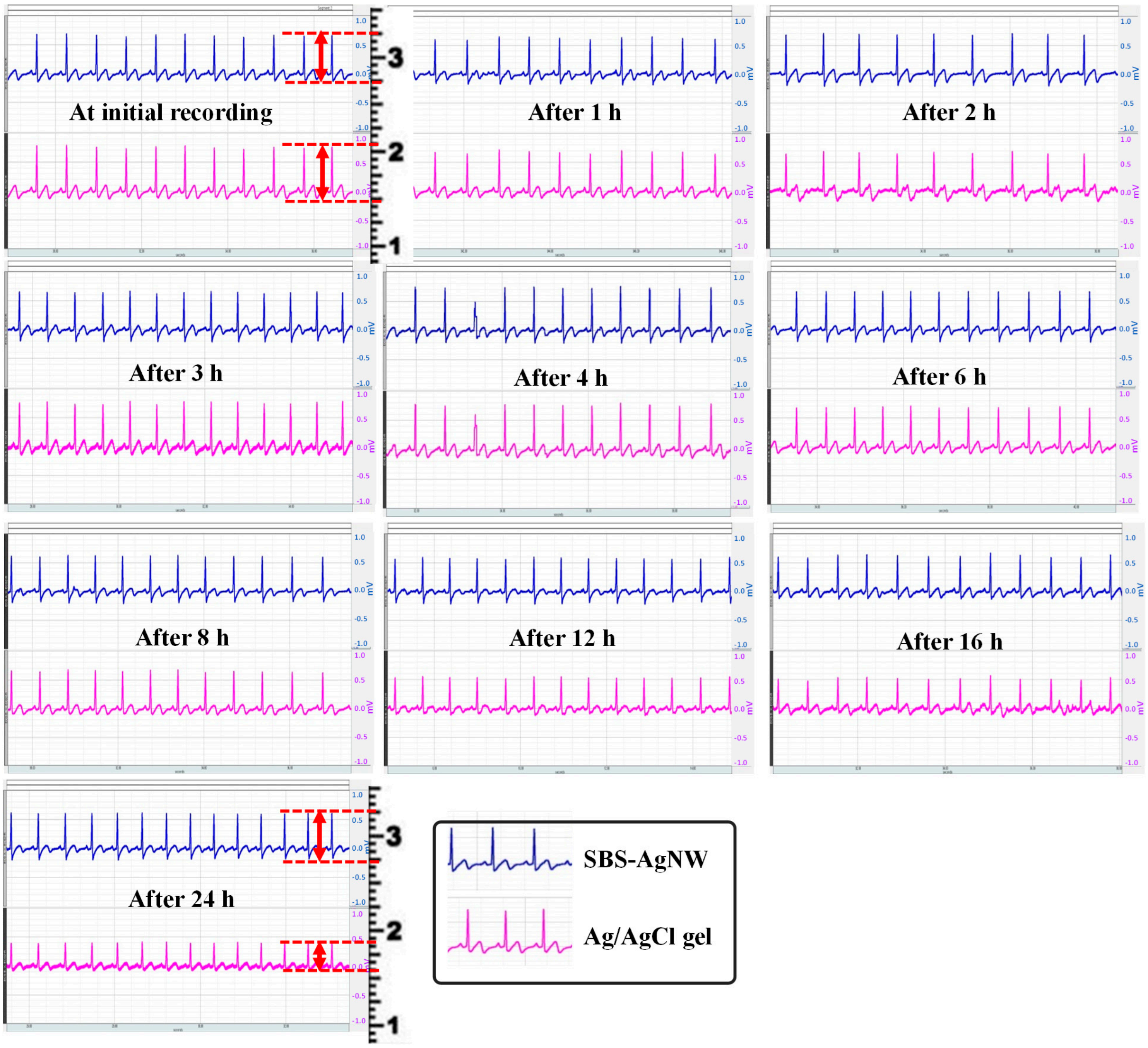

Figure S5. Screenshot image of simultaneously recorded ECG signals for SBS-AgNW dry electrode (blue) and Ag/AgCl gel electrode (pink) under various time intervals. Both the electrodes were continuously attached to the subjects' chests for a long 24 h and performed ECG recording initially after attaching the electrode, after 1 h, 2 h, 3 h, 4 h, 6 h, 8 h, 12 h, 16 h, and 24 h.

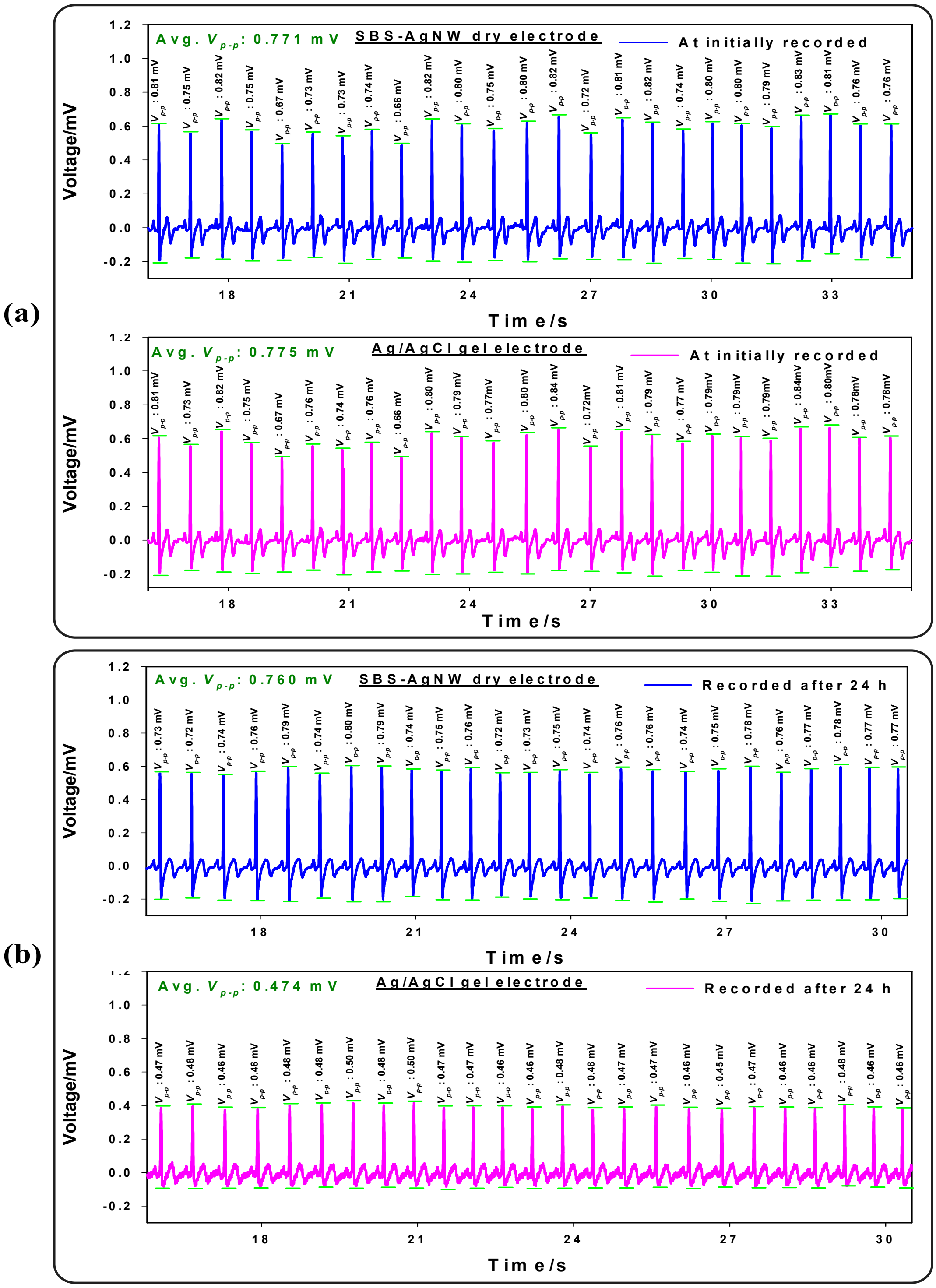

Figure S6. Peak-to-peak voltage (V_{p-p}) of about 25 ECG pulses and their average V_{p-p} for SBS-AgNW dry electrode (blue), Ag/AgCl gel electrode (pink) at the initially recorded signals **(a)**, and the signals recorded after 24 h continuously attached electrodes to the subjects' chest **(b)**.

References

(1) McAdams, E. T.; Jossinet, J.; Lackermeier, A.; Risacher, F. Factors Affecting Electrode-Gel-Skin Interface Impedance in Electrical Impedance Tomography. *Med. Biol. Eng. Comput.* **1996**, *34*, 397–408.

(2) McAdams, E. T; Jossinet. The importance of electrode-skin impedance in high resolution electrocardiography. *Automedica*, **1991**, *13*, pp. 187–208.

(3) Oh, T. I.; Koo, W.; Lee, K. H.; Kim, S. M.; Lee, J.; Kim, S. W.; Seo, J. K.; Woo, E. J. Validation of a multi-frequency electrical impedance tomography (mfEIT) system KHU Mark1: Impedance spectroscopy and time-difference imaging. *Phys. Meas.* **2008**, 29, pp. 295-307.

(4) Salvo, P.; Raedt, R.; Carrette, E.; Schaubroeck, D.; Vanfleteren, J.; Cardon, L. A 3D Printed Dry Electrode for ECG/EEG Recording. *Sens. Actuator A Phys.* **2012**, *174*, 96–102.

(5) Shyamkumar, P.; Rai, P.; Oh, S.; Ramasamy, M.; Harbaugh, R.E.; Varadan, V. Wearable Wireless Cardiovascular Monitoring Using Textile-Based Nanosensor and Nanomaterial Systems. *Electronics* **2014**, *3*, 504-520.

(6) Nicolas-Alonso, L. F.; Gomez-Gil, J. Brain Computer Interfaces, A Review. *Sensors* **2012**, *12*, 1211–1279.
